# Species distribution and antifungal susceptibility profiles of *Trichosporon* species: a multicenter study of 223 clinical isolates from South China

**DOI:** 10.3389/fcimb.2026.1982885

**Published:** 2026-10-23

**Authors:** Yangyang Qiao, Jiarong Zhan, Kaixuan Yuan, Jiankai Deng, Yaqin Peng, Huilin Yang, Shiqin Yang, Bingshao Liang, Danping Qiu, Lanping Zhang, Wenying Cai, Yanfang Wang, Penghao Guo

**Affiliations:** 1 Department of Clinical Laboratory Medicine, The First Affiliated Hospital of Sun Yat-sen University, Guangzhou, China; 2 Department of Laboratory Medicine and Institute of Precision Medicine, The First Affiliated Hospital of Sun Yat-sen University, Guangzhou, China; 3 Department of Clinical Laboratory Medicine, Guangdong Provincial People’s Hospital (Guangdong Academy of Medical Sciences), Southern Medical University, Guangzhou, China; 4 Department of Clinical Laboratory Medicine, Peking University Shenzhen Hospital, Shenzhen, China; 5 Department of Clinical Laboratory Medicine, People’s Hospital of Boluo County, Huizhou, China; 6 Department of Clinical Laboratory Medicine, Guangzhou Women and Children’s Medical Center, Guangzhou Medical University, Guangzhou, China; 7 Department of Clinical Laboratory Medicine, The First People’s Hospital of Yulin, Yulin, China; 8 Department of Clinical Laboratory, Heyuan Hospital of Traditional Chinese Medicine, Heyuan, China; 9 Department of Dermatology, Sun Yat-sen Memorial Hospital, Sun Yat-sen University, Guangzhou, China; 10 Department of Laboratory Medicine, Zhujiang Hospital, Southern Medical University, Guangzhou, China

**Keywords:** antifungal susceptibility testing, ERG11, intergenic spacer 1, MALDI-TOF MS, *Trichosporon*, whole-genome sequencing

## Abstract

**Background:**

*Trichosporon* species have emerged as opportunistic pathogens, causing life-threatening invasive infections. However, epidemiological data from South China remain limited. This multicenter study investigates the species distribution, genotypic characteristics, and antifungal susceptibility profiles of clinical *Trichosporon* isolates from this region.

**Methods:**

A total of 223 unique clinical *Trichosporon* isolates were collected from 18 centers in five South China provinces. These isolates were identified using matrix-assisted laser desorption/ionization time-of-flight mass spectrometry (MALDI-TOF MS) and sequencing of the intergenic spacer 1 (*IGS1*) region. The *Trichosporon asahii* isolates were further analyzed through *IGS1* sequence analysis for genotyping. The minimum inhibitory concentrations (MICs) of six antifungal agents were determined using the broth microdilution method outlined by the Clinical and Laboratory Standards Institute (CLSI). Whole-genome sequencing (WGS) was performed on isolates with high fluconazole MICs and on randomly selected low-MIC isolates to characterize *ERG11* mutations.

**Results:**

*IGS1* sequencing identified 220 of the 223 isolates to the species level, representing eight species, with *T. asahii* predominating (171, 77.7%). The species-level concordance between MALDI-TOF MS and *IGS1* sequencing was 85.5% (188/220), with substantial interspecies variation. Genotypes 1 (32.7%) and 3 (32.2%) co-predominated among *T. asahii* isolates, with no significant association found between genotype and specimen source (P = 0.663). Voriconazole was the most active agent, with a MIC_90_ of 0.125 μg/mL and a geometric mean (GM) of 0.06 μg/mL. High fluconazole MICs were consistently associated with specific *ERG11* substitutions: Y133F in *T. dohaense* and L229I, V132A, and Y133F in *T. asteroides.*

**Conclusions:**

*T. asahii* is the predominant clinical *Trichosporon* species in South China. Voriconazole is the most effective antifungal agent against *Trichosporon*. Species-specific *ERG11* polymorphisms may be associated with elevated fluconazole MICs.

## Introduction

The *Trichosporon* genus belongs to the phylum Basidiomycota, class Tremellomycetes, order *Trichosporon*ales, and family *Trichosporon*aceae (Miranda et al., 2025). Ubiquitous in natural environments such as soil, water, and decaying organic matter, they also colonize the human gastrointestinal tract, oral cavity, and skin as commensals (Mehta et al., 2021; Lima and Dias, 2024). Clinically, these emerging pathogens cause a broad spectrum of disease, ranging from superficial infections to life-threatening invasive *Trichosporon*osis manifesting as fungemia, urinary tract infection, peritonitis, pneumonia, or meningitis (Colombo et al., 2011; Nobrega de Almeida et al., 2021). Mortality rates for invasive disease remain exceedingly high, particularly when antifungal therapy is delayed (Francisco et al., 2019; He et al., 2025).

Over the past two decades, *Trichosporon* infections have been increasingly recognized as a significant cause of invasive fungal disease in immunocompromised hosts, including patients with hematological malignancies, solid-organ transplant recipients, and individuals with prolonged neutropenia (Chitasombat et al., 2012). *Trichosporon* has also been implicated in a growing number of breakthrough infections during echinocandin or amphotericin B prophylaxis (Matsue et al., 2006; Bayramoglu et al., 2008; Kimura et al., 2022).

Among the clinically relevant species, *T. asahii* predominates worldwide, accounting for 68–84% of invasive infections (Guo et al., 2019; de Souza Jimenez et al., 2025), followed by *T. inkin*, *T. asteroides*, *T. faecale*, *T. dermatis*, *T. mucoides*, and *T. dohaense* (Ruan et al., 2009; Yu et al., 2018). Accurate species-level identification is clinically critical, as antifungal susceptibility profiles differ markedly among species (Yu et al., 2018). However, conventional phenotypic methods frequently yield inconclusive results, and although MALDI-TOF MS offers a rapid alternative, its accuracy depends substantially on database completeness. Consequently, sequence analysis of the *IGS1* region remains the reference standard for both species identification and genotyping (Sugita et al., 2002).

Antifungal treatment of invasive *Trichosporon*osis remains challenging. *Trichosporon* species are intrinsically resistant to echinocandins, and flucytosine lacks activity against these basidiomycetous yeasts. Among the azoles, voriconazole demonstrates the most potent *in vitro* activity and is recommended as first-line therapy by the 2021 global guideline for the management of rare yeast infections (Chen et al., 2021). However, the emergence of azole-resistant *T. asahii* strains, particularly following prolonged azole exposure, poses a growing clinical threat (Padovan et al., 2019). Proposed resistance mechanisms include *ERG11* mutations, efflux-pump over-expression, biofilm formation, and activation of stress-response pathways (Iturrieta-González et al., 2014; Passos Lima et al., 2025; Shimizu et al., 2026), although whether *ERG11* variants constitute the principal driver of azole resistance remains under debate.

Moreover, substantial geographic variation has been documented in the species distribution, genotypes, and antifungal susceptibility patterns of *Trichosporon* (Xia et al., 2012; Yang et al., 2013; Guo et al., 2019). Comprehensive epidemiological data from South China are still limited, and the regional distribution of species, patterns of resistance, and correlations between genotypes and susceptibility have not been systematically characterized. To address these gaps, this multicenter study characterized 223 clinical *Trichosporon* isolates from South China. We aimed to define their species distribution, genotypic characteristics, and susceptibility patterns to inform clinical management and empirical treatment strategies.

## Materials and methods

### Study design

This retrospective study included 223 unique *Trichosporon* isolates consecutively collected from clinical specimens at 18 centers across five provinces in South China between January 2020 and December 2025. Among these isolates, 203 isolates (91.0%) were contributed by 13 centers in Guangdong Province, while the remaining 20 isolates (9.0%) were collected from 5 centers in Guangxi, Hunan, Yunnan, and Fujian provinces. As the study aimed to characterize the epidemiology of clinical isolates, no distinction was made between isolates from invasive infections and those representing colonization. All isolates were transported to the First Affiliated Hospital of Sun Yat-sen University for centralized identification and susceptibility testing.

### MALDI-TOF MS identification

All isolates were subcultured on Sabouraud dextrose agar at 35 °C for 48 h and identified using the Vitek MS system with IVD Knowledgebase v3.2 (bioMérieux). Briefly, a single colony was spotted directly onto a target plate and overlaid with 0.5 μL of formic acid; after air-drying at room temperature, α-cyano-4-hydroxycinnamic acid (CHCA) matrix was added. A confidence value of 99.9% was required for acceptable species-level identification.

### Molecular identification and *IGS1* genotyping

Genomic DNA was extracted using the Yeast Genomic DNA Rapid Extraction Kit (Sangon Biotech, Shanghai, China) according to the manufacturer’s instructions. Species-level identification was performed by sequence analysis of the *IGS1* region using primers 26SF and 5SR, as previously described (Sugita et al., 2002). All the aligned sequences were then compared with the sequence database available in GenBank by NCBI Blastn search. Genus or species level identification was based on sequence similarity thresholds: ≥99% for species assignment, 95–98% for genus assignment, and <95% for family assignment (Bosshard et al., 2003).

*T. asahii* isolates were genotyped by aligning *IGS1* sequences with reference strains retrieved from GenBank (genotypes 1-9: AB066383.1, AB072606.1, AB066397.1, AB066399.1, AB066401.1, AB180192.1, AB180194.1, AB439002.1, and AB439003.1). Sequence alignment and phylogenetic analyses were performed using MEGA version 12.0. Phylogenetic trees were constructed using the neighbor-joining method, with tree robustness evaluated by bootstrap analysis (1,000 replicates).

### Antifungal susceptibility testing

Susceptibility testing was performed for six antifungal agents, including fluconazole (FLU), voriconazole (VOR), posaconazole (POS), isavuconazole (ISV), itraconazole (IZ), and amphotericin B (AMB). All antifungal agents were purchased from Shanghai Aladdin Bio-Chem Technology Co. (Shanghai, China). Testing procedures and interpretation of results followed the CLSI M27 (4th ed.) (CLSI, 2017) and M27M44S (3rd ed.) (CLSI, 2022) documents. Quality control was maintained by parallel testing of the reference strains *Candida parapsilosis* ATCC 22019 and *Candida krusei* ATCC 6258. According to previous studies (Montoya et al., 2018; Francisco et al., 2019), *Trichosporon* isolates with a fluconazole MIC of ≥8 μg/mL were defined as high-MIC strains.

### Whole-genome sequencing and assembly

Isolates with a fluconazole MIC of ≥8 μg/mL, together with randomly selected isolates with low fluconazole MICs, were subjected to WGS. Genomic DNA was sheared to ~350 bp using a Covaris LE220R-plus (Covaris, USA). Sequencing libraries were prepared through end repair, A-tailing, adapter ligation, and PCR amplification, and purified with AMPure XP beads (Beckman Coulter, Beverly, MA, USA). Library quality was assessed on an Agilent 5400 system (AATI). Qualified libraries were pooled and sequenced on an Illumina platform with paired-end 150-bp (PE150) chemistry.

Raw sequencing reads were trimmed using Trim Galore (v0.6.10) to remove adapters and low-quality bases. For *T. asahii*, filtered reads were mapped to the reference genome of strain CBS2479 (GenBank accession no. NW_014040921.1) using BWA-MEM2 (v2.3). Because no public reference genomes are available for *T. asteroides* and *T. dohaense*, a *de novo* assembly strategy was adopted to generate custom reference genomes: clean reads of the susceptible isolates Ta-WT1 and Td-WT1 were assembled using SPAdes (v4.2.0) (Prjibelski et al., 2020) with the “--careful” parameter. Assembly quality was assessed using QUAST (v5.3.0) (Mikheenko et al., 2023) and BUSCO (v6.0.0) (Tegenfeldt et al., 2025) against the tremellomycetes_odb12 dataset. For the newly assembled genomes, gene prediction was performed with Funannotate (v1.8.17), followed by functional annotation using eggNOG-mapper (v2.1.14) (Cantalapiedra et al., 2021).

### Read mapping and variant calling

Clean reads of each isolate were aligned to the corresponding reference genome (*T. asahii* to the CBS2479 genome; *T. asteroides* and *T. dohaense* to the *de novo* assemblies Ta-WT1 and Td-WT1, respectively) using BWA-MEM2 (v2.3). PCR duplicates were marked and removed using the MarkDuplicates function of Picard (v2.20.4). Only samples with a mean depth ≥10× and a mapping rate ≥90% were retained for downstream analysis. Joint variant calling for each species was performed using FreeBayes (v1.3.10), and the resulting variants were filtered with the vcffilter function of vcflib (v1.0.14) (Garrison et al., 2022) under the following criteria: QUAL > 30, DP > 5, SAF > 0, SAR > 0, RPR > 1, RPL > 1, and MQM > 40. Functional annotation of the identified variants was conducted using SnpEff (v5.3.0a) (Cingolani et al., 2012) with locally built databases for each species.

### Statistical analysis

Statistical analyses were performed using WHONET 5.6, Microsoft Excel, and SPSS 25.0. For each species–agent combination, the MIC range, MIC_50_, MIC_90_, and GM MIC were calculated. The association between genotype and specimen type, both categorical variables, was assessed using the Pearson chi-square test. When expected frequencies were below five, the likelihood-ratio test and a sensitivity analysis merging the genotypes with small counts were additionally performed. A two-sided *P* value < 0.05 was considered statistically significant.

## Results

### Clinical and demographic characteristics

The study population was predominantly male (162, 72.6%; male-to-female ratio, 2.7:1). Patient ages ranged from 0 to 101 years (mean, 55.8 years; median, 59.0 years), with a bimodal distribution peaking in the 51–60 and 61–70 year age groups (**Figure 1A**). Most isolates were obtained from intensive care units (74, 33.2%), followed by surgical (57, 25.6%), internal medicine (51, 22.9%), and hematology departments (19, 8.5%). The remaining 22 isolates (9.9%) were recovered from other departments (**Figure 1B**). By specimen type, urine was the most common source (70, 31.4%), followed by sputum or bronchoalveolar lavage fluid (BALF) (52, 23.3%) and secretions (32, 14.3%). Blood cultures yielded 16 isolates (7.2%), and the remaining 53 isolates (23.8%) came from various other sources (see **Figure 1C**).

**Figure 1:**
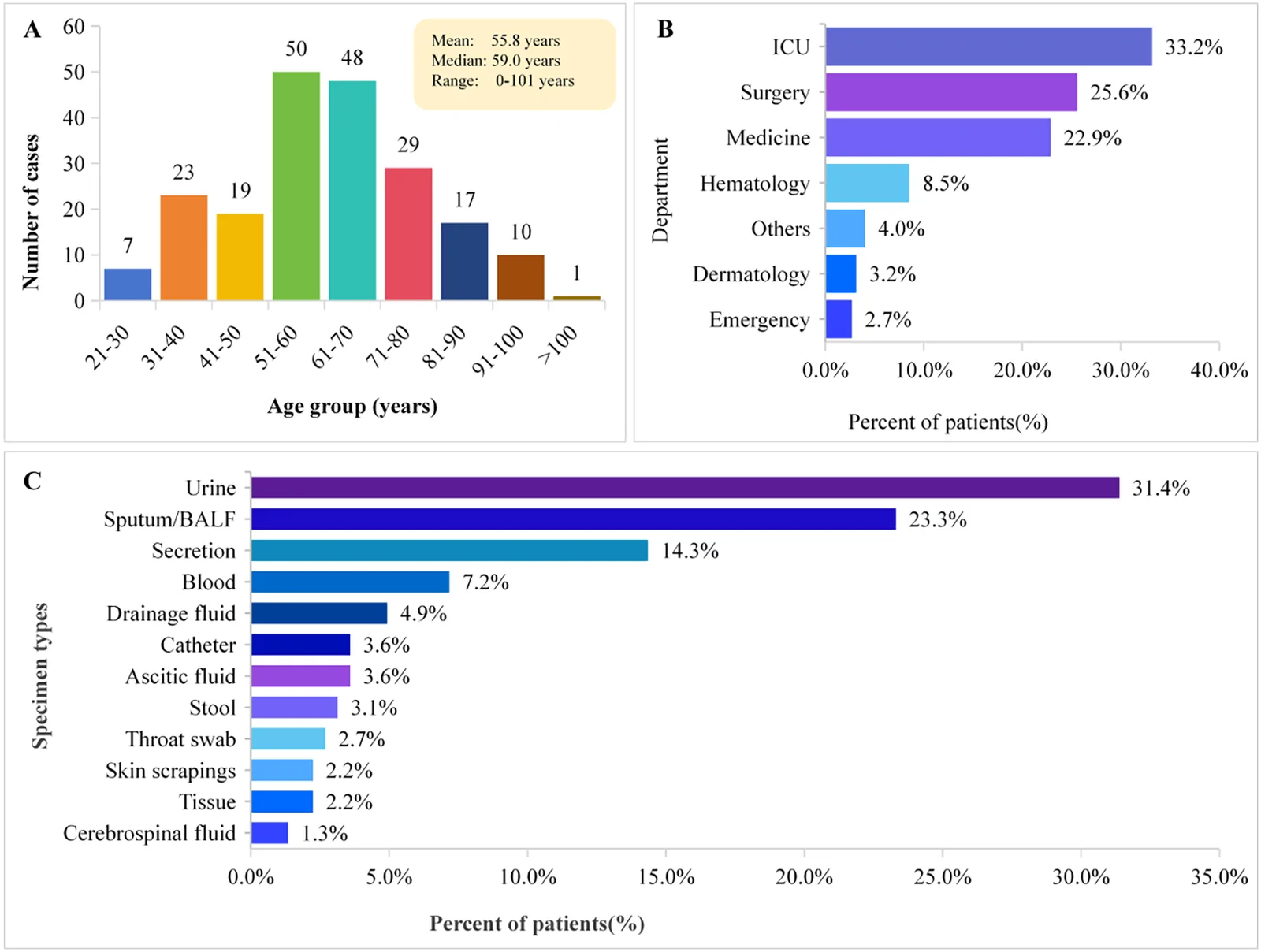
Demographic and clinical characteristics of the study population. **(A)** Age distribution of the 223 patients. The histogram shows the number of cases in each age group. **(B)** Distribution of patients by hospital department. The bar chart illustrates the percentage of patients admitted to different departments, with ICU (33.2%), Surgery (25.6%), and Medicine (22.9%) being the most common. **(C)** Distribution of clinical specimen types. The bar chart displays the percentage of isolates recovered from various sources, with urine (31.4%), sputum/BALF (23.3%), and secretion (14.3%) accounting for the majority of samples.

### Species distribution and MALDI-TOF MS identification accuracy

*IGS1* sequencing identified 220 of the 223 isolates to the species level. The remaining three isolates showed sequence similarities below the 99% threshold required for species-level assignment and were therefore designated *Trichosporon* spp. (genus level only). The 220 isolates identified to the species level comprised eight *Trichosporon* species, with *T. asahii* predominating (171, 77.7%), followed by *T. faecale* (15, 6.8%), *T. asteroides* (10, 4.5%), *T. dohaense* (9, 4.1%), *T. dermatis* (7, 3.2%), *T. inkin* (3, 1.4%), *T. terricola* (3, 1.4%), and *T. jirovecii* (2, 0.9%).

Among these 220 isolates, MALDI-TOF MS identified 188 to the species level and two (one *T. terricola* and one *T. faecale*) to the genus level only, while the remaining 30 yielded no identification. Accordingly, the species-level concordance between MALDI-TOF MS and *IGS1* sequencing was 188/220 (85.5%). Concordance was complete for *T. asahii*, *T. asteroides*, and *T. dermatis*, whereas no isolates of the remaining species (*T. faecale*, *T. dohaense*, *T. inkin*, *T. terricola*, and *T. jirovecii*) were correctly identified to species level.

### *IGS1* genotype distribution of *T. asahii*

The 171 *T. asahii* isolates were classified into six genotypes (see **Figure 2**). Genotype 1 (56/171, 32.7%) and genotype 3 (55/171, 32.2%) codominated, followed by genotype 4 (41/171, 24.0%), genotype 2 (10/171, 5.8%), and genotypes 5 (5/171, 2.9%) and 6 (4/171, 2.3%). The distribution of genotypes across specimen types is shown in **Table 1**. Because frequencies were below 5, the likelihood-ratio test and a sensitivity analysis merging genotypes 5 and 6 were performed in addition to the Pearson chi-square test. No statistically significant association was observed between genotype and specimen type (χ² = 16.850, df = 20, *P* = 0.663; likelihood-ratio G² = 16.773, *P* = 0.668; after merging genotypes 5 and 6, χ² = 16.720, df = 16, *P* = 0.404).

**Figure 2:**
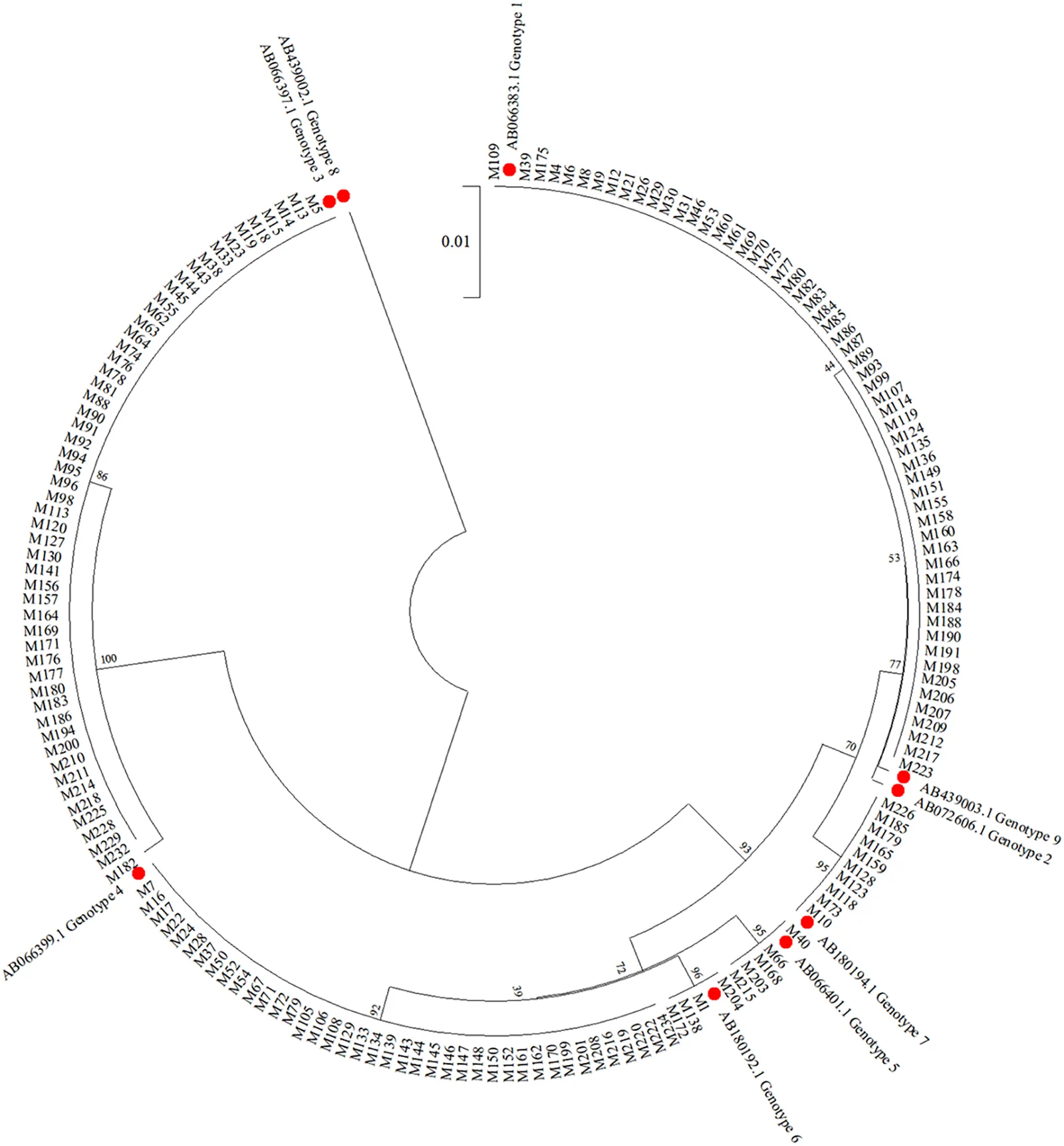
Genotype distribution of *T. asahii* based on *IGS1* sequences. Circular neighbor-joining phylogenetic tree constructed from the *IGS1* sequences of 171 *T. asahii* clinical isolates and nine genotype reference sequences retrieved from GenBank, using the Kimura 2-parameter model in MEGA 12.0. The scale bar represents 0.01 nucleotide substitutions per site. Reference sequences are marked with red dots.

**Table 1:** Distribution of *T. asahii* genotypes by specimen source (n = 171).

Genotype	Urine	Sputum/BALF	Secretion	Blood	Others	Total
Genotype 1	17 (30.4%)	13 (23.2%)	8 (14.3%)	6 (10.7%)	12 (21.4%)	56
Genotype 2	2 (20.0%)	2 (20.0%)	4 (40.0%)	0 (0.0%)	2 (20.0%)	10
Genotype 3	23 (41.8%)	11 (20.0%)	6 (10.9%)	3 (5.5%)	12 (21.8%)	55
Genotype 4	13 (31.7%)	13 (31.7%)	6 (14.6%)	2 (4.9%)	7 (17.1%)	41
Genotype 5	1 (20.0%)	3 (60.0%)	0 (0.0%)	0 (0.0%)	1 (20.0%)	5
Genotype 6	1 (25.0%)	2 (50.0%)	0 (0.0%)	0 (0.0%)	1 (25.0%)	4
**Total**	**57 (33.3%)**	**44 (25.7%)**	**24 (14.0%)**	**11 (6.4%)**	**35 (20.5%)**	**171**

Data are presented as n (% of the corresponding genotype total). BALF, bronchoalveolar lavage fluid.

Bold values indicate the total number of *T. asahii* isolates for each specimensource column.

### Antifungal susceptibility patterns

The *in vitro* activities of six antifungal agents against all 223 isolates were evaluated. The MIC ranges, MIC_50_, MIC_90_, and GM MICs are summarized in **Table 2**. Among the 223 *Trichosporon* isolates, voriconazole demonstrated the strongest *in vitro* activity, with an MIC_50_ of 0.06 μg/mL, an MIC_90_ of 0.125 μg/mL, and a GM of 0.06 μg/mL. This was followed by posaconazole (GM, 0.10 μg/mL), isavuconazole (GM, 0.11 μg/mL), and itraconazole (GM, 0.15 μg/mL). Fluconazole had the highest overall MICs, with an MIC_90_ of 4 μg/mL and a GM of 2.70 μg/mL. In comparison, amphotericin B had an MIC_90_ of 2 μg/mL and a geometric mean MIC of 0.83 μg/mL.

**Table 2:** *In vitro* antifungal susceptibilities of 223 clinical *Trichosporon* isolates by the CLSI broth microdilution method.

Species	FLU	VOR	POS	ISV	IZ	AMB
All isolates (n = 223)						
MIC range	0.125–256	0.015–8	0.004–1	0.008–2	0.015–1	0.25–4
MIC50	2	0.06	0.125	0.125	0.125	1
MIC90	4	0.125	0.25	0.25	0.5	2
GM	2.70	0.06	0.10	0.11	0.15	0.83
*T. asahii* (n = 171)						
MIC range	0.125–16	0.015–0.5	0.015–0.5	0.015–1	0.015–0.5	0.25–4
MIC50	2	0.06	0.125	0.125	0.125	1
MIC90	4	0.125	0.25	0.25	0.5	2
GM	2.58	0.06	0.11	0.12	0.16	0.88
*T. faecale* (n = 15)						
MIC range	1–4	0.015–0.06	0.008–0.25	0.015–0.25	0.03–0.25	0.25–1
MIC50	2	0.03	0.06	0.06	0.125	1
MIC90	4	0.06	0.25	0.125	0.125	1
GM	1.91	0.04	0.07	0.06	0.09	0.76
*T. asteroides* (n = 10)						
MIC range	1–256	0.03–8	0.06–1	0.06–2	0.06–1	0.5–2
MIC50	2	0.06	0.06	0.25	0.125	1
MIC90	256	4	0.5	2	1	1
GM	12.13	0.32	0.15	0.43	0.25	0.93
*T. dohaense* (n = 9) ^a^						
MIC range	1–32	0.03–0.5	0.015–0.25	0.03–1	0.03–0.125	0.25–2
MIC50	2	0.06	0.125	0.125	0.125	0.5
MIC90	32	0.5	0.25	1	0.125	2
GM	4.32	0.10	0.08	0.21	0.08	0.58
*T. dermatis* (n = 7) ^a^						
MIC range	2–4	0.03–0.125	0.03–0.06	0.03–0.125	0.03–0.125	0.25–0.5
MIC50	2	0.06	0.06	0.06	0.06	0.5
MIC90	4	0.125	0.06	0.125	0.125	0.5
GM	2.69	0.06	0.05	0.07	0.07	0.45
*T. inkin* (n = 3)						
MIC range	1–2	0.03–0.06	0.03–0.25	0.008–0.125	0.06–0.25	0.5–2
*Trichosporon spp*. (n = 3)						
MIC range	2–4	0.03–0.06	0.03–0.06	0.06–0.125	0.03–0.125	0.25–1
*T. terricola* (n = 3)						
MIC range	1–1	0.03–0.03	0.06–0.06	0.03–0.06	0.06–0.06	1–1
*T. jirovecii* (n = 2)						
MIC range	0.5–4	0.015–0.03	0.004–0.015	0.015–0.06	0.03–0.03	0.25–0.5

All MIC values are in μg/mL. GM, geometric mean. MIC_50_ and MIC_90_, the lowest MICs inhibiting ≥50% and ≥90% of isolates, respectively. FLU, fluconazole; VOR, voriconazole; POS, posaconazole; ISV, isavuconazole; IZ, itraconazole; AMB, amphotericin B.

^a^ Species with 5 < n < 10 isolates; the corresponding MIC_50_/MIC_90_ and GM values are descriptive only and should be interpreted with caution.

*T. asteroides* showed the highest azole MICs among all species, with fluconazole MIC_90_ of 256 μg/mL, followed by voriconazole (4 μg/mL), isavuconazole (2 μg/mL), itraconazole (1 μg/mL), and posaconazole (0.5 μg/mL). *T. dohaense* also exhibited elevated fluconazole MICs, with an MIC_90_ of 32 μg/mL. In contrast, *T. faecale* and *T. dermatis* consistently displayed low MICs across all antifungal agents. The remaining three species (*T. inkin*, *T. terricola*, and *T. jirovecii*) also showed low MICs, but due to the small number of isolates for these species (n ≤ 3), only the MIC distributions were reported.

### Association between *ERG11* mutations and fluconazole MIC

Using whole-genome sequencing data, we analyzed the association between *ERG11* gene sequences and the fluconazole MIC in *T. asahii*, *T. asteroides*, and *T. dohaense*.

Phylogenetic analysis was performed using the *ERG11* coding sequences of five sequenced *T. asahii* isolates (four with high fluconazole MICs and one with a low MIC) and the reference strain CBS2479 (fluconazole MIC, 2 μg/mL) (Nobrega de Almeida et al., 2022). The phylogenetic tree resolved the six sequences into two distinct clades (**Figure 3A**): clade A (Tas17, Tas141, Tas46) and clade B (Tas164, the reference strain CBS2479, Tas200). Notably, each clade contained both isolates with high fluconazole MICs and isolates with low MICs, indicating that the *ERG11* phylogenetic background was not associated with the high-MIC phenotype. Furthermore, the *ERG11* exon sequences were 100% identical between the high-MIC strain Tas17 and the low-MIC strain Tas141, as well as between the high-MIC strains Tas200 and Tas164 and the low-MIC reference strain CBS2479. The antifungal MICs of the five sequenced isolates and CBS2479 are shown in **Figure 3B**.

**Figure 3:**
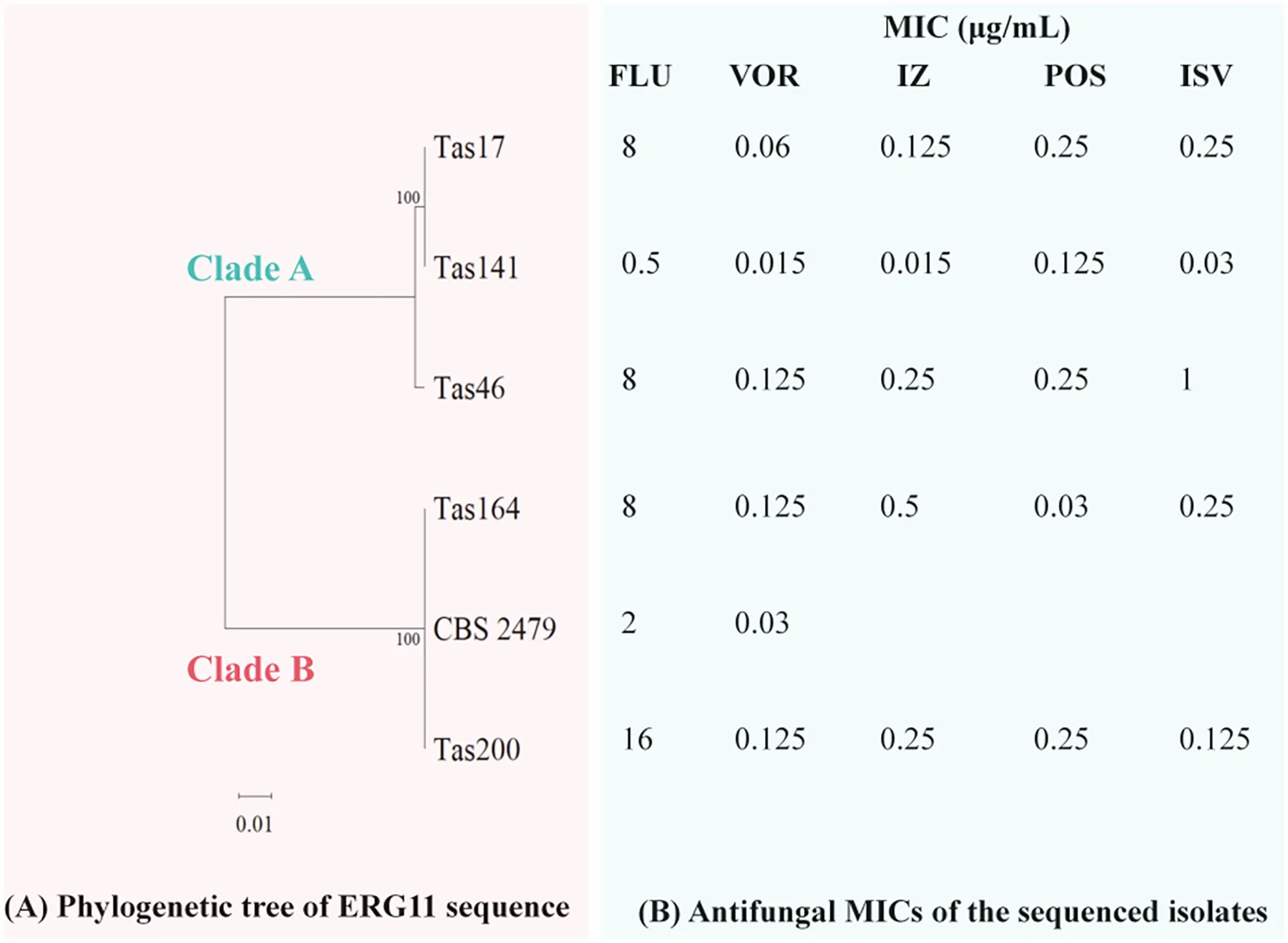
Association between *ERG11* phylogeny and antifungal susceptibility in *T. asahii*. **(A)** Phylogenetic tree based on the *ERG11* sequences of the five sequenced *T. asahii* isolates and the reference strain CBS2479. The tree was constructed using the neighbor-joining method with the Kimura 2-parameter model (bootstrap 1,000 replicates) in MEGA 12.0. The scale bar indicates 0.01 nucleotide substitutions per site. Clade A comprises Tas17, Tas141, and Tas46; clade B comprises Tas164, CBS2479 and Tas200. **(B)** MICs (μg/mL) of the corresponding isolates against five antifungal agents. Isolates with fluconazole MICs ≥8 μg/mL were classified as high-MIC; CBS2479 was not tested in this study and is shown as low-MIC based on its reported fluconazole susceptibility. FLU, fluconazole; VOR, voriconazole; POS, posaconazole; ISV, isavuconazole; IZ, itraconazole.

For *T. asteroides* and *T. dohaense*, we analyzed four high fluconazole MIC isolates of each species, with fluconazole MICs ranging from 128 to 256 µg/mL and 8 to 32 µg/mL, respectively. Additionally, two control isolates for each species, each with a fluconazole MIC of 1 µg/mL, were included. Since no public reference genomes were available for these two species, we generated *de novo* assemblies for the susceptible isolates Ta-WT1 and Td-WT1 to serve as references. This allowed us to analyze the mutational landscape of the *ERG11* gene. All four high fluconazole MIC isolates of *T. asteroides* consistently carried three amino acid substitutions in *ERG11*: L229I (c.685C>A), and the tandem V132A and Y133F substitutions arising from a multi-nucleotide variant (c.395_398delTGTAinsCGTT). In contrast, the Y133F substitution (c.398A>T) was consistently present in all four high fluconazole MIC *T. dohaense* isolates. The co-occurrence of L229I with the tandem V132A/Y133F substitutions in *T. asteroides* may account for its markedly higher fluconazole MICs relative to *T. dohaense*. The *ERG11* mutations and antifungal MICs (μg/mL) of *T. asteroides* and *T. dohaense* isolates are shown in **Table 3**.

**Table 3:** *ERG11* mutations and antifungal MICs (μg/mL) of the *T. asteroides* and *T. dohaense* isolates.

Isolate	Species	*ERG11* mutation(s)	FLU	VOR	POS	ISV	IZ
Ta-M1	*T. asteroides*	L229I; V132A; Y133F	128	1	0.25	0.25	0.25
Ta-M2	*T. asteroides*	L229I; V132A; Y133F	256	4	1	2	0.5
Ta-M3	*T. asteroides*	L229I; V132A; Y133F	256	4	0.5	2	1
Ta-M4	*T. asteroides*	L229I; V132A; Y133F	256	8	0.5	2	1
Ta-WT1	*T. asteroides*	None	1	0.03	0.06	0.25	0.125
Ta-WT2	*T. asteroides*	None	1	0.03	0.06	0.06	0.06
Td-M1	*T. dohaense*	Y133F	16	0.25	0.25	1	0.125
Td-M2	*T. dohaense*	Y133F	16	0.25	0.125	0.5	0.125
Td-M3	*T. dohaense*	Y133F	8	0.25	0.125	0.5	0.125
Td-M4	*T. dohaense*	Y133F	32	0.5	0.25	1	0.125
Td-WT1	*T. dohaense*	None	1	0.06	0.06	0.125	0.06
Td-WT2	*T. dohaense*	None	1	0.03	0.03	0.03	0.125

None: indicates no mutations were detected. FLU, fluconazole; VOR, voriconazole; POS, posaconazole; ISV, isavuconazole; IZ, itraconazole.

## Discussion

To our knowledge, this multicenter study represents the largest species-level characterization of clinical *Trichosporon* isolates from South China to date. It provides a comprehensive analysis of this emerging basidiomycetous pathogen, covering its species distribution, genotypic characteristics, and antifungal susceptibility profiles. Furthermore, it describes the association between elevated fluconazole MICs and *ERG11* substitutions.

Our cohort showed a marked male predominance and a bimodal age distribution peaking at 51–70 years, consistent with multicenter studies from Brazil and Saudi Arabia (Nobrega de Almeida et al., 2021; Almarhabi et al., 2025). The high proportions of isolates from intensive care units and surgical departments suggest an association between *Trichosporon* isolation and the healthcare setting. This distribution aligns with the common risk factors for nosocomial colonization or infection, such as prolonged hospitalization, invasive procedures, and broad-spectrum antimicrobial exposure (Liao et al., 2015; Nobrega de Almeida et al., 2021). Urine was the most common specimen source, consistent with findings from a cohort at Anhui Medical University, in which urine accounted for 76.8% of isolates (Xu et al., 2026). This predominance of urinary specimen isolation may reflect the propensity of *Trichosporon* species to form biofilms on indwelling urinary catheters, thereby promoting colonization and subsequent infection in hospitalized patients (Lara et al., 2023).

Accounting for 77.7% of the isolates, *T. asahii* was the predominant species, consistent with its status among clinical *Trichosporon* isolates worldwide (Francisco et al., 2019; Kuo et al., 2021; Xu et al., 2026). In total, eight species were identified, including the rarely reported *T. terricola*, *T. inkin*, and *T. jirovecii*. In addition, three isolates could only be assigned to the genus level and were designated *Trichosporon* spp. These isolates may represent unrecognized *Trichosporon* genetic diversity in South China. Their precise taxonomic status cannot be resolved by *IGS1* sequencing alone and warrants confirmation by sequencing of a second locus (e.g., the *ITS* region or 26S rDNA *D1/D2*) and, ideally, whole-genome analysis in future studies.

Most of the isolates in this study were obtained from non-sterile sites, so the species distribution and antifungal susceptibility data reflect the overall characteristics of “clinical isolates” rather than just those from invasive infections. However, the species distribution of isolates from sterile sites (n=40), including blood, ascites, tissue, and drainage fluid, was largely consistent with the overall cohort, which was predominantly *T. asahii*. This indicates that, despite certain limitations, the overall data still provides valuable insights for assessing invasive infections.

MALDI-TOF MS achieved 100% concordance for the three species (*T. asahii*, *T. asteroides*, and *T. dermatis*), whereas none of the isolates belonging to the remaining five species was identified to the species level. Consistent with previous findings, the Hefei study reported that both the bioMérieux and DL platforms performed well for *T. asahii* but poorly for non–*T. asahii* species (Xu et al., 2026). The inability to identify less common species is closely associated with incomplete database coverage for rare taxa. The Vitek MS IVD Knowledgebase v3.2 contains reference spectra for only a limited number of *Trichosporon* species, and the absence or poor representation of spectra for less common species such as *T. faecale*, *T. dohaense*, and *T. inkin* accounts for their identification failures. Therefore, isolates that cannot be confidently identified by MALDI-TOF MS, particularly non–*T. asahii* isolates, should be subjected to sequencing-based identification.

In contrast to the predominance of genotype 1 reported from Spain, Mexico, Brazil, and Japan (Sugita et al., 2002; Rodriguez-Tudela et al., 2007; Chagas-Neto et al., 2009; Montoya et al., 2015), genotypes 1 and 3 codominated in our cohort, a pattern more similar to that reported from Taiwan, Thailand, and India, where genotypes 1 and 3 are both prevalent (Mekha et al., 2010; Yang et al., 2013; Rastogi et al., 2016). The absence of an association between genotype and specimen type suggests that the major *T. asahii* genotypes share similar niches and that no single genotype is preferentially associated with a particular specimen source.

As reported previously, azoles showed potent *in vitro* activity against *Trichosporon* (Guo et al., 2019). The geometric mean MIC ranking (voriconazole > posaconazole ≈ isavuconazole > itraconazole > amphotericin B > fluconazole) aligns with the 2021 global guideline recommending voriconazole as a preferred first-line therapy for trichosporonosis (Chen et al., 2021). Furthermore, due to considerable inter-strain variability in antifungal susceptibility results within the same *Trichosporon* species, routine susceptibility testing is essential. This study did not include susceptibility testing for echinocandins, as *Trichosporon* species inherently resist this class of antifungal agents. Consequently, breakthrough Trichosporonosis should be considered in patients receiving caspofungin or micafungin as prophylaxis (Chen et al., 2021).

Although the underlying mechanisms remain incompletely understood, elevated fluconazole MICs in *Trichosporon* have been associated with biofilm formation, *ERG11* mutations, and efflux pump overexpression (Iturrieta-González et al., 2014; Mehta et al., 2021; Ma et al., 2023). Among the reported mechanisms, *ERG11* mutations have garnered particular attention. For instance, Kushima H et al (Kushima et al., 2012). demonstrated that the G453R substitution in *ERG11* of *T. asahii* confers high fluconazole MICs. Additionally, Abbes et al. (2021) identified potential associated mutations, including H380G, S381A, and H437R. However, some studies indicate that *ERG11* variants in *T. asahii* isolates are not the primary determinant of high-MIC phenotypes (Nobrega de Almeida et al., 2022). Consistent with previous reports, we did not find any exonic polymorphisms in the *ERG11* gene among *T. asahii* isolates with either high or low fluconazole MICs. Additionally, the high-MIC isolates were found across both phylogenetic clades. These findings suggest that mechanisms other than *ERG11* mutations—such as efflux pump overexpression or biofilm formation—may play a more prominent role in *T. asahii* (Kushima et al., 2012; Abbes et al., 2021; Nobrega de Almeida et al., 2022).

Tyr133 in *Trichosporon ERG11* is notably equivalent to the well-known azole-resistance hotspot Tyr132 in *Candida albicans ERG11* (Walsh et al., 1990; Kushima et al., 2012; Kushima et al., 2017). The Y133F substitution was consistently present in all high-MIC *T. dohaense* isolates, and Y133F/V132A/L229I in all high-MIC *T. asteroides* isolates. Given the known role of the Tyr132-equivalent residue in azole resistance in *C. albicans*, these substitutions are plausible candidates contributing to the high fluconazole MICs observed in these species. However, since our sample size was small and selected based on MIC results, these data demonstrate an association rather than a direct cause-and-effect relationship. Functional validation of these findings is necessary.

In this multicenter study from South China, *T. asahii* was the predominant clinical *Trichosporon* species, and *IGS1* genotypes 1 and 3 codominated. Voriconazole demonstrated the strongest *in vitro* activity, confirming its effectiveness as a first-line therapy. While MALDI-TOF MS proved to be reliable for identifying common species, it was inadequate for rare species, which still require sequencing-based identification for accurate diagnosis. Species-specific *ERG11* substitutions—Y133F in *T. dohaense* and L229I, V132A, and Y133F in *T. asteroides*—were consistently linked to high fluconazole MICs.

Several limitations of this study should be acknowledged. First, as colonization and infection were not distinguished, the data reflect the overall clinical isolate population rather than strains causing invasive infections. Second, the uneven geographic distribution of isolates (91.0% originating from Guangdong Province) precluded stratified analyses by province. Third, while *ERG11* variants were screened via whole-genome sequencing, functional assays to validate their causal role and alternative resistance pathways were not assessed. Thus, the identified *ERG11* substitutions represent candidate resistance markers rather than confirmed mechanisms, warranting future functional studies.

## Data Availability

The datasets presented in this study can be found in online repositories. The names of the repository/repositories and accession number(s) can be found in the article/supplementary material.
